# Diverse Host Plants of the First Instars of the Invasive *Lycorma delicatula*: Insights from eDNA Metabarcoding

**DOI:** 10.3390/insects13060534

**Published:** 2022-06-10

**Authors:** Cameron McPherson, Alina Avanesyan, William O. Lamp

**Affiliations:** Department of Entomology, University of Maryland, College Park, MD 20742, USA; cmcpher@terpmail.umd.edu (C.M.); lamp@umd.edu (W.O.L.)

**Keywords:** eDNA metabarcoding, host-plant usage, insect gut content, invasive species, spotted lanternfly, trophic interactions

## Abstract

**Simple Summary:**

The globalization of human activities, especially in agriculture, has facilitated the range expansion of insect pests, promoting species invasions in new territories. Here, we focused on the invasive spotted lanternfly, *Lycorma delicatula*, an important economic pest, accidentally introduced in the United States and first detected in 2014. Our study investigated host-plant usage by the first nymphal instars, which are more challenging to find and monitor than other stages. Using DNA metabarcoding of nymphal gut contents (i.e., detection of DNA from multiple ingested plants) in this study we determined and characterized the ingested plants that could be included in a broad host-plant range of early nymphal stages of *L. delicatula*. This, in turn, will have important applications for developing effective management programs to control the invasive spotted lanternfly. The results from our study will be of great interest for all the forest managers and growers in the potential (national) range of the spotted lanternfly, and will help them ultimately minimize their efforts and expenses needed for managing this important pest.

**Abstract:**

Identification of host plants of the invasive spotted lanternfly, *Lycorma delicatula* (Hemiptera: Fulgoridae), has been the focus of many studies. While the adults and late nymphs are relatively easy to observe on plants and to use for molecular gut-content analysis, studying the early instars is more challenging. This study is the continuation of our ongoing efforts to determine the host range for each developmental stage of *L. delicatula*. In the present study, we focused exclusively on the first nymphal instars, and we used a novel approach, utilizing “bulk” DNA extracts for DNA metabarcoding of nymphal gut contents, to identify all the detectable plants that the nymphs had ingested prior to being collected. We were able to obtain high-quality amplicons (up to 406 bp) of a portion of the *rbc*L gene and detect 27 unique ingested plant species belonging to 17 families. Both native and introduced plants with the prevalence of trees and grasses were present among the ingested plants. We also identified 13 novel host plants that have not been previously reported for *L. delicatula* on the U.S. territory. The results from our study have important applications for developing effective programs on early monitoring of invasive *L. delicatula*.

## 1. Introduction

The spotted lanternfly, *Lycorma delicatula* (Hemiptera: Fulgoridae), is a highly invasive sap-feeding insect in North America, and one of the most aggressive insect pests in the Mid-Atlantic region of the United States [1,2,3,4,5,6,7]. Since its first discovery in Berks County, Pennsylvania, from Asia in 2014, *L. delicatula* has quickly dispersed and established its populations in neighboring states and beyond. The invasion of *L. delicatula* continues on U.S. territory: to date, *L. delicatula* individuals have been detected in 15 states; of these, 9 states have heavy infestations, and 4 states have multiple internal quarantine zones [8].

The host-plant range of *L. delicatula* has been the focus of many studies as it is highly polyphagous, and current host lists span a large number of host plants of various taxonomic levels, life forms, morphology, and plant architecture, as well as both coevolved (i.e., originated in Asia) and noncoevolved hosts (i.e., originated in other regions than Asia, both native and introduced to the U.S.), including various forest trees (tree-of-heaven, birch, maple, walnut, oak, etc.), fruit trees (such as apple trees and grapes), as well as many ornamental plants [4,7,9,10,11]. Being highly polyphagous, *L. delicatula* poses a significant risk to forestry and agriculture; adults and four nymphal instars feed on host phloem tissues, causing plant injury including plant photosynthesis reduction, weeping wounds, and creating conditions for sooty mold that ultimately affects plant growth and reduces the fruit quality [1,3,5,6,12].

Management, and especially early monitoring of *L. delicatula*, is challenging, particularly due to its unusual use of plant hosts during the insect development; as the nymphs mature, their host plant range decreases, and by the time the insects reach the adult stage they have one or two preferred host plants [1]. To date, there are multiple ongoing efforts to decipher the host-plant range of *L. delicatula* at both the adult stage and nymphal stages with successful attempts to accurately confirm the consumed host plants by adults and late nymphal instars [9]. Early nymphal instars, however, and especially the first instar, received less attention in the experimental studies. Due to their small size, cryptic coloration, and a discreet sap-feeding behavior, the first instars are challenging to find and monitor. While most studies on *L. delicatula* feeding preferences still focus on the experimental observations [5,13], recent advances using PCR-based DNA analyses provide opportunities for rapid and reliable detection and identification of *L. delicatula*’s diet [6,14]. In our previous work on plant DNA detection from the gut contents of *L. delicatula* [6], we focused on late nymphal instars only (third and fourth nymphal instars). Using Sanger sequencing to detect the most abundant plant item in the lanternfly gut contents, we found that (a) ingested plants in ~93% of the nymphs did not correspond with the plants from which the nymphs were collected (possibly due to high mobility of nymphs), and (b) both coevolved (i.e., introduced into the U.S.) and noncoevolved (i.e., native to the U.S.) plants, as well as woody and nonwoody plants, were ingested. Additionally, to optimize our DNA work, we conducted a systematic review of published PCR approaches to detect the host plants ingested by insect pests [15].

DNA metabarcoding of insect gut contents, in particular, has been demonstrated as an effective approach that allows us to accurately determine a host-plant range of insect pests [16], to reconstruct the invasion route of insect pests [17], insect pest migration [18,19,20], as well as to record realized and novel plant–insect associations [21,22,23]. In general, the advantage of DNA metabarcoding over observation or morphology-based approaches to insect diet analysis is detection of ingested plant diversity and composition [24]. The DNA metabarcoding approach has important applications for biomonitoring of species in diverse communities [25], and is particularly applied to restoration monitoring [24]. Additionally, recent practices in environmental DNA (eDNA) barcoding (i.e., simultaneous identification of species from multiple taxa within one environmental sample, using a specific fragment of DNA) demonstrated the advantage of using environmental bulk DNA extracts over DNA barcoding of individual organisms [26,27,28]. Following these recent advances, in this study, for the first time, we apply a “bulk” extraction of DNA from multiple nymphal individuals of *L. delicatula*. We expected that this approach (a) would be beneficial for increasing DNA yield in each bulk sample (compared to a “single-insect” approach); (b) would be time- and cost-efficient; and most importantly, (c) would result in diverse gut-content samples which, in turn, would accurately represent the diversity of ingested plant species, which is the primary focus of our study. Finally, following suggested applications in Van Der Heyde et al. [24], we aimed to show the utility of the DNA metabarcoding of gut contents of *L. delicatula* in early monitoring of the first nymphal instars, for which there is a growing need. Such a DNA metabarcoding approach is necessary for effective monitoring of movement of *L. delicatula* populations on their host plants, as well as early detection of novel host plants and potential host switch.

To address these goals, the main focus of this study was to demonstrate the utility of eDNA metabarcoding for determining a host-plant range for the first nymphal instar of the invasive spotted lanternfly, *L. delicatula*. Additionally, we explore the diversity of ingested plants in terms of woodiness and perenniality, as well as the proportions of coevolved/noncoevolved host plants. We also expected to detect the ingested plant species that had not been previously recorded as host plants of *L. delicatula*. Based on these findings, we discussed important implications for potential host plants of early nymphal instars and their effective monitoring. Since we focused on the ingested plants only, similar to our previous research on diet analysis of insect pests [6,29], and for the purpose of this study only, we will continue using the term “host plants” to indicate consumption of insect food plants only, i.e., the plants that *L. delicatula* nymphs utilize as suitable food source, and not especially suitable as sources for reproduction and development.

## 2. Materials and Methods

### 2.1. Study Site

**Figure 1:**
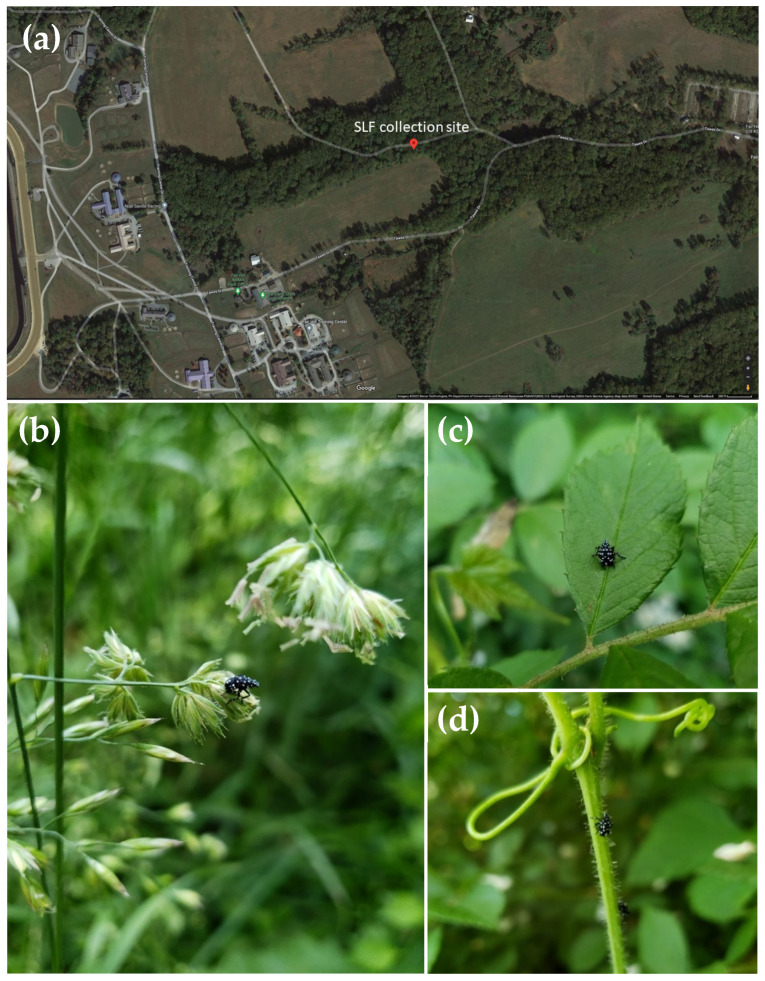
Location of the collection site at the Fair Hill State Natural Resource Management Area (39°42′36.3′′ N, 75°51′02.98′′ W, Elkton, MD, USA) (**a**), and first nymphal instars of *Lycorma delicatula* observed and collected from various plants (**b**–**d**) (Photos by Alireza Shokoohi).

The plants and the first nymphal instars were collected in Cecil County (MD, USA) in May 2021 during two collection trips, a week apart. The collection site (30 × 73 m^2^) was a forested area adjacent to a grass field and was located in the Fair Hill State Natural Resource Management Area (FH-NRMA) (39°42′36.3′′ N, 75°51′02.98′′ W, Elkton, MD, USA) (Figure 1a). The weather conditions at the site, on both collection days, ranged from no clouds to partial coverage with a temperature between 26–29 °C and calm-to-light wind. This site was chosen due to (a) the known established population of *L. delicatula* in this area; (b) a high number of observed egg masses on tree trunks; and (c) its high plant diversity (especially in woody plants). The FH-NRMA is located in Fair Hill, northeastern MD, in a 12 ha forested area. Previous studies have indicated that the area contains sites with high tree density (225 trees per ha), relatively high tree-canopy height (27.8 m on average), and is dominated by *Fagus grandifolia* (American beech), *Liriodendron tulipifera* (yellow poplar), *Acer rubrum* (red maple), *Betula lenta* (sweet birch) and *Quercus alba* (white oak) [30], all of which were reported as preferred or suitable host plants of *L. delicatula* [9].

### 2.2. Sample Collection and Processing

In this study, a total of 37 first nymphal instars of *L. delicatula* and 28 reference plants (i.e., one clipped portion and one leaf sample per unique plant growing at the collection site) were collected from plants along the margin between the grass field and the forested area (Figure 1b–d). That was also the only location the nymphs were observed; all the nymphs were observed on woody plants facing south. The plant samples were collected from the collection site only (and not from the entire Fair Hill State Natural Resource Management Area), i.e., from the site where the nymphs were observed and collected. Of the 28 plant samples collected, *L. delicatula* nymphs were only observed and collected from four plants: *Rosa* sp., *Rubus phoenicolasius*, *Ailanthus altissima*, and *Celastrus orbiculatus* (Table 1; samples p001, p002, p005, and p007). Sequence analysis of DNA extracted from the collected plants showed 19 unique plant species. After collection, nymphs and leaf samples were immediately dry-frozen at the site and transported to our laboratory at the University of Maryland where they remained stored at −20 °C until DNA extraction. The clipped plant samples were used to create herbarium samples to aid morphological plant identification when necessary.

### 2.3. DNA Extraction, PCR Amplification, and Purification

For this study, we utilized our previously developed approach for identification of ingested plants from the gut contents of *L. delicatula* [6], the potato leafhopper *Empoasca fabae* [29], and earlier *Melanoplus* grasshoppers [31,32]. The entire body of *L. delicatula* nymph and 5–10 mm sized cuts of plant tissue from the leaf samples were used for DNA extraction. To prepare bulk samples, anywhere from six to ten nymphs at a time (depending on insect size) were placed in a 1.5 mL microcentrifuge tube (Fisher Scientific Co., Pittsburg, PA, USA) and their bodies were ground up using RNase-free disposable pellet pestle (Fisher Scientific Co., Pittsburg, PA, USA). The resulting tissue mixture was then evenly separated into other 1.5 mL microcentrifuge tubes, approximately 100 mL into each, to satisfy the manufacturer protocol for DNA extraction. Genomic DNA of the *L. delicatula* nymph bulk samples was extracted using the DNeasy Blood and Tissue kit (catalog no. 69506, Qiagen Inc., Germantown, MD, USA) following the manufacturer’s protocol. Once DNA was isolated, the samples were stored at −4 °C until PCR amplification. In addition, DNA from one individual first nymphal instar was also extracted; this was used as a reference sample.

On the next step of sample processing, genomic DNA extracts from both individual sample and bulk samples were used to detect a portion (~530 bp) of a coding region of the chloroplast DNA, *rbc*L gene (ribulose-1,5-biphosphate carboxylase-oxygenase). Primers rbcLaF and rbcLaR (purchased from Integrated DNA Technologies, Inc., San Diego, CA, USA) were used to amplify the targeted DNA region. PCR was run following the protocol described in Avanesyan and Lamp [6] using PCR components and conditions indicated in Table 2. PCR products were then purified using Exo-SAP-IT (catalog no. 78201.1.ML, Affymetrix Inc., Santa Clara, CA, USA) according to manufacturer’s protocol. Leaf samples were processed following Avanesyan et al. [29]; same portion of the *rbc*L gene was amplified from each DNA extract, and purified, followed by Sanger sequencing which was conducted at Azenta/GENEWIZ (Azenta US, Inc., South Plainfield, NJ, USA).

### 2.4. Sequence Analysis

The obtained PCR products from insect samples containing amplified plant regions were used for sequencing. Next-generation sequencing (“Amplicon-EZ” service), followed by unique sequence abundance analysis, were conducted at Azenta/GENEWIZ (Azenta US, Inc., South Plainfield, NJ, USA). Amplicon-EZ service provides sequencing and analysis of heterogeneous PCR products. This service processes amplicons up to 500 bp and produces 50,000+ reads per sample. This service was chosen due to (a) full coverage the amplicons of the portion of the rbcL-gene, which was utilized for DNA barcoding; and (b) interactive analysis report of detected unique sequences that can be conveniently used for subsequent species identification. Obtained raw reads were trimmed and merged fasta-files were generated at Azenta using an in-house script; forward and reverse primers (described above) were then used to generate the final unique consensus sequences for any DNA region nested by the pair of these primers. Analysis of sequence quality was conducted on raw reads, for each bulk sample, using FastQC tool in Galaxy platform [33].

Plant species identity for both insect samples (i.e., ingested plants) and plant samples (i.e., reference plants) was determined using BLAST engine in the National Center for Biotechnology Information (NCBI) GenBank database (https://www.ncbi.nlm.nih.gov/genbank/, accessed on 7 November 2021–3 April 2022). The unique ingested plant species were then determined across all the bulk samples of *L. delicatula* nymphs. For leaf samples, one forward and one reverse sequence were obtained from Azenta; the sequences were then trimmed using 4Peaks v. 1.7 and aligned using Unipro UGENE platform. Each consensus sequence was then used to determine the plant identity using BLAST, the NCBI GenBank database (also accessed on 7 November 2021–3 April 2022).

Sequence reads obtained from the gut contents of *L. delicatula* nymphs were sorted by sequence length, and all the sequences longer than 100 bp were used for plant identification. Of these, sequences that showed >90% in matches were used for further analysis. This sequence length was chosen based on our preliminary work on sequence identity, during which the sequences shorter than 100 bp demonstrated a low percentage of identity (50–70%) in matches with sequences deposited in the NCBI GenBank database. Plant origin in relation to North America, and specifically to the eastern US (native vs. introduced), as well as plant life form of all identified ingested plant species was determined using the USDA Plant database (https://plants.usda.gov/home, accessed on 7 November 2021–3 April 2022).

### 2.5. Measurements and Statistical Analysis

The obtained data on the presence of various ingested plant species in the gut contents of *L. delicatula* nymphs were first synthesized using counts and proportions. For the reference samples and each bulk sample, a list of unique identified ingested plants with corresponding sequences was first compiled (please see Supplementary Material). Next, a combined list of unique ingested plants across all the samples was constructed, and proportions of various plant families, as well as plants with different origin and life form were calculated. Additionally, for the bulk samples, a proportion of each unique plant species was determined. The prevalence of plant species of different origin, family, as well as various life forms, was analyzed using an exact binomial test. For the purpose of this study, the null hypothesis used for the binomial test was that the types of plants (i.e., woody vs. nonwoody, etc.) were represented in the gut contents of *L. delicatula* nymphs in equal proportions.

Mean quality scores for each group of forward and reverse-sequence reads were retrieved using FastQC tool in Galaxy platform; only the scores for sequences longer than 100 bp (for the bulk samples) and 150 bp (for the reference samples) were included in data analysis. Mean quality scores were then compared among all the samples using a one-way ANOVA with a post hoc TukeyHSD. The Shapiro–Wilk and Bartlett tests were used to investigate the normality and heteroscedasticity of data, respectively. Data analysis, followed by creating pie charts and a boxplot, was conducted in R v.4.1.0 [34].

## 3. Results

### 3.1. Basic Sequence Statistics

**Figure 2:**
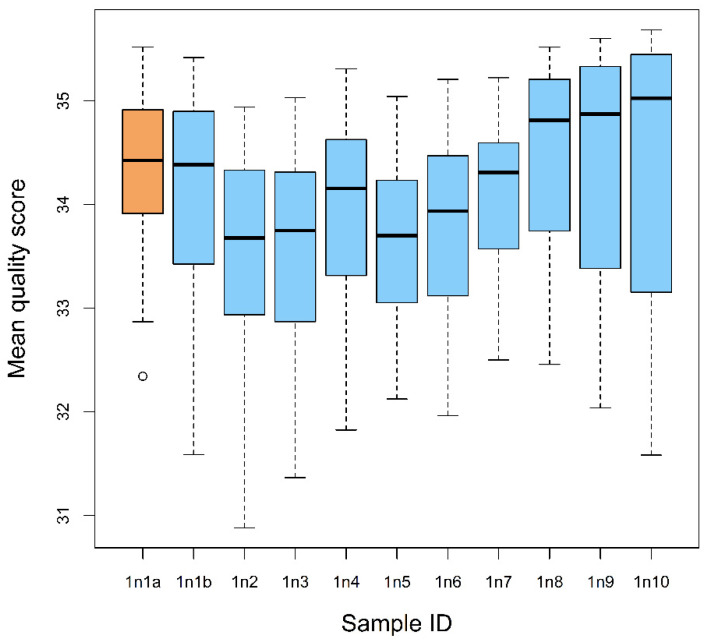
Mean quality scores of the readable sequences obtained from each bulk sample and from the reference sample of gut contents of the first nymphal instars of *Lycorma delicatula*. Sample “1n1a” indicates the reference sample; samples 1n1b-1n10 are the bulk samples.

On average, 170,078 ± 9680 total sequence reads were retrieved from each bulk sample (samples 1n1-1n10) and 347,901 reads were obtained from a reference sample (one individual nymph) (Table 3). For the bulk samples, the analysis of consensus sequences revealed 6.9 ± 1.7 readable sequences longer than 100 bp, with the average quality scores >33 (high sequence quality); 65 readable sequences, 150 bp and longer, were obtained from the reference sample (Table 3, Figure 2).

We did not observe any pattern of sequence quality scores across the reference and bulk samples: three bulk samples showed the mean sequence scores that were significantly lower than that in the reference sample (ANOVA: F10,651 = 9.475, *p* < 0.001; TukeyHSD: *p* < 0.05), while the differences in the mean quality scores between the reference sample and other bulk samples were not significant. Similarly, pairwise comparisons of the bulk samples (46 pairs total) showed significant difference in the mean quality in 15 pairs, while the scores in the other pairs were not significantly different (Table 3).

### 3.2. Diversity of Ingested Plants

**Figure 3:**
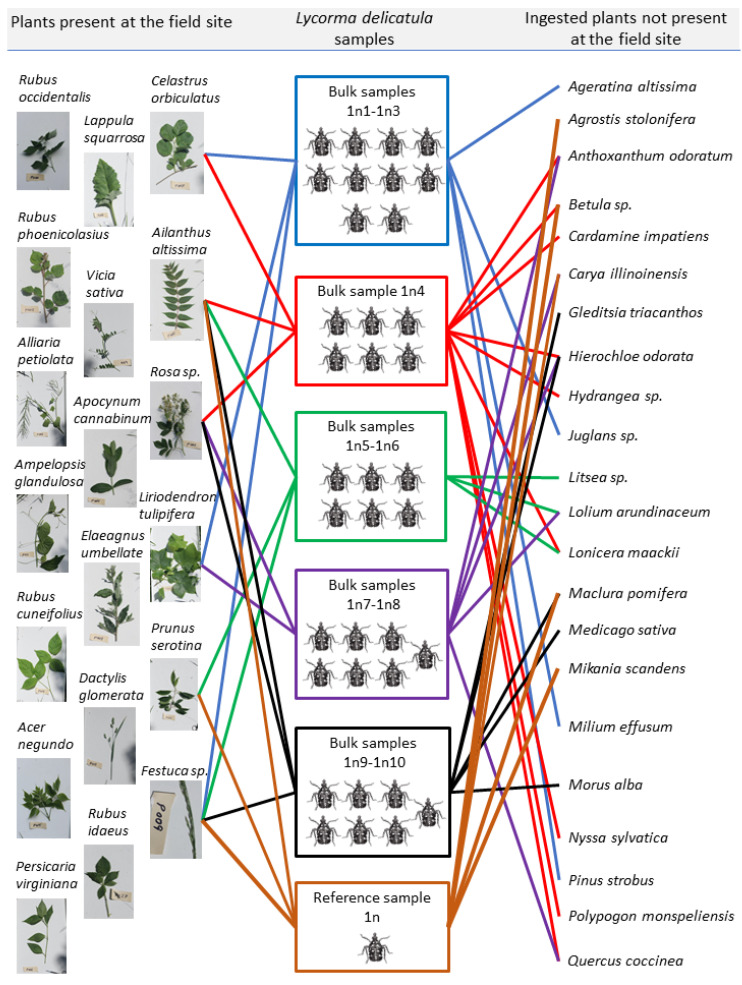
The species identity of the ingested plants obtained from both bulk samples and reference sample of the gut contents of the first nymphal instars of Lycorma delicatula. The lines show the trophic interactions between the nymphs and plants that were present at the field site (DNA detected; on the left: 6 out of 19 plants at the site) and the plants that were absent at the field site (on the right). The plants that were present at the field site refer to the plants that were present at the collection site where the insect nymphs were collected, and not to the plants growing in the Fair Hill State Natural Resource Management Area (Elkton, MD, USA). Each box with L. delicatula nymphs shows a separate group of nymphs; the number of nymphs in each box corresponds to the number of nymphs which were ground together to create bulk DNA extracts. The bulk sample labels show the number of bulk samples obtained from each group of nymphs (e.g., 1n1-1n3 represent three bulk samples created from 10 nymphs). Trophic interactions are shown and were analyzed for each group of nymphs separately (i.e., each line of a different color coming from a different box represents separate nymphs), and then the results on ingested plants were summarized to include all the bulk samples and the reference sample. (Photos of the herbarium samples by W. Lamp; drawings of the nymphs by A. Avanesyan; the chart design by C. McPherson).

Sequence analysis of DNA extracted from the collected plants showed 19 unique plant species (Figure 3). DNA metabarcoding of gut contents of 37 first nymphal instars of L. delicatula revealed 27 unique ingested plant species; of these, six plant species were present at the collection site, two of which were the plants the nymphs were collected from (Table 4, Figure 3). From one to 12 unique plant species were identified from the bulk samples (samples 1n1-1n10; 4 ± 1.01 plant species per sample), and 11 unique plant species were identified from the reference sample, with *Ailanthus altissima* and *Festuca sp*. being the most common plants detected across all the samples (Figure 3). Interestingly, sample “1n4” containing a bulk DNA extract from six nymphs yielded the maximum number (12) of the unique ingested plants. Sequences from all the identified plant species demonstrated 99–100% (Mean% ± SE: 99.79 ± 0.06) match with sequences for corresponding plant species deposited in the NCBI GenBank database (Table 4).

Of the 27 unique ingested plants, 22 plants were identified to species and 5 plants to genus, with 2–3 best matches. The latter included the following genera: *Betula* sp. (with the highest match for *Betula lenta*, *Betula pendula*, and *Betula papyrifera*); *Hydrangea sp.* (high match with multiple species); *Festuca* sp. (with the highest match for *Festuca brevipila* and *Festuca ovina*); *Litsea* sp. (high match with multiple species); *Prunus* sp. (with the highest match for *Prunus serotina* and *Prunus virginiana*), and *Rosa* sp. (with the highest match for *Rosa laevigata*, *Rosa multiflora*, and *Rosa rugosa*). The presence of all of these identified ingested plant species in Cecil County, Maryland, USA was confirmed through the USDA PLANT database (https://plants.usda.gov/java/, accessed on 3 April 2022). Finally, when the list of identified ingested plants was compared with the latest published host plant list for *L. delicatula* [9], and with the recent findings on ingested plants from *L. delicatula* gut contents [14], 13 novel species/genera were identified (Table 4).

**Figure 4:**
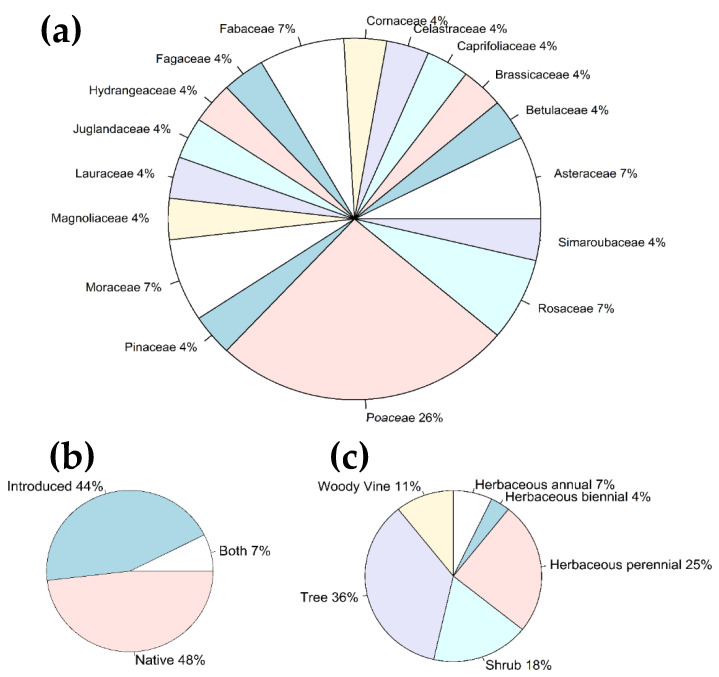
Ingested plant species, obtained from both bulk samples and reference sample of the gut contents of the first nymphal instars of Lycorma delicatula. (**a**) Overall proportion of unique plant families; (**b**) overall proportion of ingested plants with different plant origin (“Both” represents *Betula* sp. and *Hydrangea* sp.); and (**c**) overall proportion of ingested plants with various life forms.

**Figure 5:**
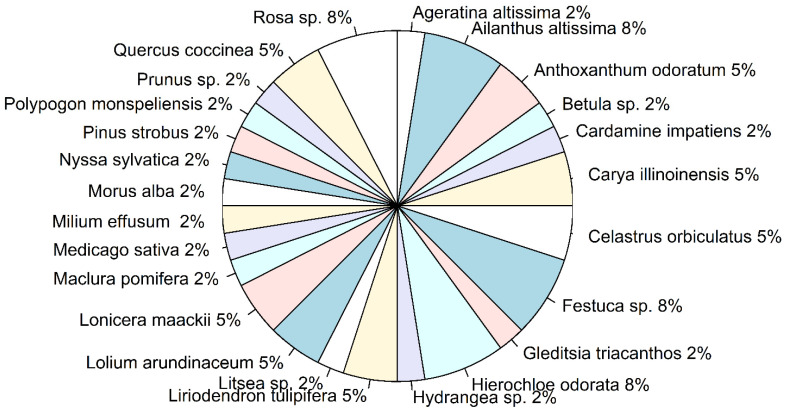
Ingested plant species, obtained from the bulk samples only. The pie chart shows the proportion of unique plant species across the bulk samples shown on Figure 2 which represented separate groups of *Lycorma delicatula* nymphs (i.e., “1n1-1n3”, “1n5”, “1n5-1n6”, “1n7-1n8”, and “1n9-1n10). The percentage of plant species was calculated across unique plant species identified for each group of bulk samples.

Identified ingested plants, from both the reference and bulk samples, belonged to 17 families (Figure 4a). Both native (48%) and introduced (44%) plants were identified among all the unique ingested plants, with 7% of plants (genera *Betula* sp. and *Hydrangea* sp.), which include both native and introduced species (Figure 4b). Trees and herbaceous plants were significantly prevalent among the ingested plants (Binomial test: *p* < 0.05)(Figure 4c); of all the herbaceous plants, grasses of the Poaceae family were dominant (Binomial test: *p* < 0.05). When the proportions of ingested plant species were analyzed across the bulk samples only, no significant prevalence of any of the species was observed (Binomial test: *p* > 0.05) (Figure 5).

## 4. Discussion

The results from this study demonstrated the successful effective application of eDNA metabarcoding to diet analysis of the first nymphal instars of *L. delicatula*. Amplicons of a portion of the chloroplast *rbc*L gene (up to 406bp) were reliably detected and identity of ingested plants was determined. Through the DNA metabarcoding of the gut contents of the fist nymphal instars of *L. delicatula*, we were able to (a) detect 27 unique ingested plant species belonging to 17 families; (b) demonstrate that up to 12 unique plant species can be retrieved per bulk sample containing 6–10 nymphs; (c) identify 13 novel host plants that have not been previously included in published host plant lists for *L. delicatula* in the U.S. territory; and (d) show that both native and introduced plant species with the prevalence of trees and grasses were suitable for feeding by the fist nymphal instars of *L. delicatula*. We discuss potential implications of these results for host interactions, ecology, and early monitoring of *L. delicatula* below.

### 4.1. Diversity of Ingested Plants

Our results support previous findings of polyphagous feeding behavior of early instars of *L. delicatula* [1,14,35]. Particularly, many authors reported the narrowing pattern of *L. delicatula* host plant use, from the first instars to adults [1,3,4,35,36]. Dechaine et al. [35] in their study observed first instars of *L. delicatula* on 33 different plant species; this number then decreases to 25 in late instars to 3 in adults. Nixon et al. [37] evaluated the survival of early nymphal instars on 10 host plants and showed significantly higher levels of survivorship on tree of heaven and black walnut. We detected *Juglans* sp. in one of the bulk samples, and a closely related pecan, *Carya illinoinensis*, from the same Juglandaceae family, was also commonly present in both the reference sample and bulk samples. Interestingly, even though *C. illinoinensis* was the best species match when GenBank was used for identification of the obtained sequence, the collection site was not located in the typical range of this species. It is possible that either these plants were ingested by the lanternfly nymphs while foraging at ornamental plantings near the collection site, or plant sequences for the obtained portion of the *rbc*L-gene are shared among other closely related *Carya* species.

Our findings also support, once again, the preference for tree of heaven, *Ailanthus altissima*, which was present at the collection site and was detected in 55% of all the samples. Cooper et al. [14] showed that up to 17 plant families can be detected from a combined 1st–3rd instars; of these, however, only 10 different taxa (belonging to 7 plant families) were reported for the first instars with 2.5 plant taxa per nymphal individual. The authors, however, used only three nymphal individuals and amplified portions of *trn*F and *ITS* genes. We demonstrated in our study that using a portion of *rbc*L gene in combination with bulk DNA extracts resulted in high DNA yield and detection up to 27 unique ingested plants with up to 12 unique taxa per sample. Future studies might further focus on differences in DNA yield from individual vs. bulk insect samples and optimization of eDNA metabarcoding protocols specifically for diet analysis using gut-content environmental samples.

Barringer and Ciafré [9] in their review reported a broad global host range of *L. delicatula*. We specifically used this published list to determine whether our findings could potentially contribute to and expand the host plant list of *L. delicatula*. Thirteen novel plant taxa that we reported in this study were also determined by comparison with the findings by Cooper et al. [14], the only study that used DNA metabarcoding of gut contents of the first instars of *L. delicatula*. We emphasize that these are novel food plants, and whether they support the development and reproduction of *L. delicatula* could also be a focus for future studies. Our findings of 13 novel plant taxa have especially important applications for early monitoring of *L. delicatula*; currently, the control programs heavily focus on egg mass surveys [5]. However, monitoring potential food plants near or around the location of egg masses is also critical.

Our findings of many ingested plant species that were not present at the collection site are in agreement with our previous findings during detection of plant DNA from gut contents in late nymphal instars of *L. delicatula* [6]. We support our previous interpretation of these results by high mobility of nymphs, and possibly by sampling a number of plants before staying on a plant for feeding. Pearson et al. [38] have shown that psyllids use their stylets to sample the parenchyma cells before finding and ingesting phloem sap. DNA from such parenchyma cells, then, can be detected in insect gut contents. However, the feeding choice of the first instars is especially interesting. In the eastern U.S., *L. delicatula* lays egg masses once a year, typically from September to November or even December [39]. If the egg masses are laid on the tree trunk, they may be laid up to 17 m above the ground [38]. The eggs hatch in May; once hatched from the eggs (if they are on the tree trunk), the first nymphal instars move up along the tree trunk [1]. Due to various physical forces (such as wind) the nymphs fall to the ground; within 1–2 days the nymphs would ascend the tree again [1]. Considering this cyclic behavior on a host tree, the prevalence of grasses in the nymphal gut contents could be explained by insect feeding on grasses, or at least sampling the grasses, during those days while the nymphs are on or close to the ground before they repeat ascending the tree. It would be interesting for future studies to use eDNA metabarcoding and explore plant diversity in soil samples as DNA from seeds, roots, or plant parts can also be picked up during the nymphs probing or feeding.

### 4.2. Dispersal and Feeding of L. delicatula Nymphs

Dispersal is an important factor facilitating invasion of an introduced insect; thus, understanding the patterns of the insect dispersal is critical for effective management [40]. Flight dispersal patterns have been well-explored in *L. delicatula* adults. Previous studies showed that the adult can spontaneously fly up to 50 m at an average speed of 4.64 m/s before landing on the tree trunks of any available tree [41,42,43]. Baker et al. [42] suggested that *L. delicatula* can move until they meet the food sources needed to complete their development. Similarly, an interesting study by Domingue et al. [44] showed that the adults of *L. delicatula* which flew towards the open field (potentially attracted by the wavelengths detected in ambient light), often changed their direction and flew towards the shade and towards the trees, presumably for feeding.

While all stages of *L. delicatula* can contribute to its dispersal, the first instars initiate the pest movement in the certain areas; this, in turn, contributes to *L. delicatula* chances to find suitable hosts. Little is known about the dispersal of nymphs, though Keller et al. [39] conducted a mark-release-resight study in contiguous deciduous forest at a temporal level exploring the dispersal distances up to 10 days after the release. The authors observed some of the nymphs staying near the release point, while some of the nymphs moved to a variety of trees, shrubs, and understory plants up to 65 m away by day 10. On average, the nymphs were able to move up to 10 m during a single day. For the first instars, specifically, the authors indicated the median dispersal distance as 3.2 m, and up to 6.2 m 7 days after release. It was also shown that most of the nymphs could be recapture within 10 m in the presence of *Ailanthus altissima* [45]. It would be interesting for future studies to explore feeding of nymphs during their movement away from egg masses, and to assess the plant diversity, cover, and species richness at certain distances from the primary egg masses, to then compare these findings with the data on ingested plant species retrieved from the nymphal gut contents.

### 4.3. Potential Limitation

During this study we investigated ingested food plants, so we expected the DNA of ingested plants to be degraded to some extent. In case digestion of certain plants was fully completed, we did not expect to detect the DNA from those plants within the lanternfly gut contents. Thus, based on our previous work with *L. delicatula* and other sap-feeders, we expected to find detectable plant DNA within a few hours after ingestion. As a result, the host-plant range we aimed to identify reflected the host plants ingested over the past few hours at the collection site and might potentially exclude some of the suitable (previously known or unknown) host plants of *L. delicatula*. Therefore, the 27 ingested plants identified in this study represented a “screenshot” of the plant species that had been consumed and of which remains were still present in the gut contents of nymphs at the moment of collection. Given that three dozen individuals were processed, and that the DNA barcoding process was consistent across all the samples, we believe that this limitation did not affect the overall accuracy of our findings and the outcomes will be valuable for creating a host-plant range for each developmental stage of *L. delicatula*. This potential limitation could be addressed in future studies by increasing the number of collected nymphs and variety of collection sites to account for any other potential host plants.

Another potential limitation is a possible presence of pollen grains on the insect body surface, which could be detected from the unsterilized insect body surface. We indeed cleaned the insect bodies with 2% bleach solution in our previous studies [29,32]. However, we recently found that the body-surface contamination with plant DNA in sap-feeding potato leafhoppers was not significant (Avanesyan and Lamp, *in prep.*). Based on these findings, and due to high mobility of the first nymphal instars we did not expect the insect body surface to be contaminated with significant amount of DNA from pollen. In the previous studies, however, we only used Sanger sequencing, and it is possible that the NGS technology which we used in this study was more sensitive to detect any plant DNA fragments which might be present on the insect body surface. We plan to address this issue in our future DNA barcoding studies by a detailed comparison of concentration of plant DNA obtained from gut-content samples and from the insect body surface.

## 5. Conclusions

The egg masses of *L. delicatula* are typically laid on tree trunks and branches, which might serve as the first suitable hosts for newly hatched first nymphal instars. However, multiple plants can be sampled and ingested during the movement of the nymphs around their first host plant. Our study showed that plants from at least 27 different taxa, both native and introduced, as well as plants of various life forms, can be used as food plants for the first nymphal instars at a relatively small collection site. In combination with results of previous studies on the nymphal dispersal, our findings emphasize the importance of early monitoring of *L. delicatula* nymphs, not only on plants in close proximity to the egg masses, but also within several meters from the “initial” host plants.

## Figures and Tables

**Table 1:** Identified reference plant species collected at the field site at the Fair Hill State Natural Resource Management Area (Elkton, MD, USA). Both the plants on which the nymphs of *Lycorma delicatula* were observed, and the plants that were free from infestation were collected.

Sample ID	Plant Family	Plant Species ID	Common Name	Nymphs Presence	Date Submitted to GenBank	GenBank Accession Number
p001	Rosaceae	*Rosa* sp.	Rose	+	12/27/2021	OM038103
p002	Rosaceae	*Rubus phoenicolasius*	Wine raspberry	+	12/27/2021	OM038104
p003	Elaeagnaceae	*Elaeagnus umbellata*	Autumn olive	−	12/27/2021	OM038105
p004	Apocynaceae	*Apocynum cannabinum*	Indian hemp	−	12/27/2021	OM038106
p005	Simaroubaceae	*Ailanthus altissima*	Tree of heaven	+	1/31/2022	OM470964
p006	Polygonaceae	*Persicaria virginiana*	Jumpseed	−	1/31/2022	OM470965
p007	Celastraceae	*Celastrus orbiculatus*	Oriental bittersweet	+	11/7/2021	OL539536
p008	Brassicaceae	*Alliaria petiolata*	Garlic mustard	−	11/21/2021	OL603937
p009	Poaceae	*Festuca sp.*	Fescue	−	11/24/2021	OL657222
p010	Fabaceae	*Vicia sativa*	Common vetch	−	11/24/2021	OL657223
p011	Vitaceae	*Ampelopsis glandulosa*	Amur peppervine	−	1/31/2022	OM470966
p012	Celastraceae	*Celastrus orbiculatus*	Oriental bittersweet	−	1/31/2022	OM470967
p013	Poaceae	*Dactylis glomerata*	Cat grass	−	2/4/2022	OM523099
p014	Rosaceae	*Rubus cuneifolius*	Sand blackberry	−	2/4/2022	OM523098
p015	Sapindaceae	*Acer negundo*	Boxelder maple	−	2/11/2022	OM672995
p016	Celastraceae	*Celastrus orbiculatus*	Oriental bittersweet	−	2/11/2022	OM672996
p017	Celastraceae	*Celastrus orbiculatus*	Oriental bittersweet	−	2/4/2022	OM523100
p018	Boraginaceae	*Lappula squarrosa*	Bristly sheepburr	−	2/4/2022	OM523101
p019	Poaceae	*Dactylis glomerata*	Cat grass	−	2/11/2022	OM672997
p020	Rosaceae	*Rubus occidentalis*	Black Raspberry	−	2/11/2022	OM672998
p021	Elaeagnaceae	*Elaeagnus umbellata*	Autumn olive	−	2/11/2022	OM672999
p022	Elaeagnaceae	*Elaeagnus umbellata*	Autumn olive	−	3/8/2022	OM964639
p023	Vitaceae	*Ampelopsis glandulosa*	Amur peppervine	−	2/11/2022	OM673000
p024	Sapindaceae	*Acer negundo*	Boxelder maple	−	2/11/2022	OM673001
p025	Magnoliaceae	*Liriodendron tulipifera*	Tulip tree	−	2/11/2022	OM673002
p026	Rosaceae	*Prunus serotina*	Black cherry	−	2/11/2022	OM673003
p027	Rosaceae	*Rubus idaeus*	Red raspberry	−	2/11/2022	OM673004
p028	Boraginaceae	*Lappula squarrosa*	Bristly sheepburr	−	2/11/2022	OM673005

**Table 2:** PCR components and conditions used to amplify a portion of *rbc*L gene in this study.

Primers and Other PCR Components	Concentration/Volume (for 20 μL of PCR Reaction)	PCR Conditions
*rbc*LaF (5′-ATG TCA CCA CAA ACA GAG ACT AAA GC-3′)	2 μM/2 μL	initial denaturation: 94 °C for 4 min 35 cycles of 94 °C for 30 s, 57 °C for 30 s, and 72 °C for 30 s final extension: 72 °C for 2 min
*rbc*LaR (5′-GTA AAA TCA AGT CCA CCR CG-3′)	2 μM/2 μL	
2X PCR PreMix with Dye (Syd Labs Inc., Natick, MA, USA)	10 μL	
ddH_2_O	5.2 μL	
template DNA	0.8 μL	

**Table 3:** Basic summary of sequence reads obtained from gut contents of first nymphal instars of Lycorma delicatula and used for identification of ingested plants. Both the reference sample (1n1a-ref) and bulk samples (1n1b-1n10) are included.

Sample ID	Total Number of Sequence Reads		Sequence Read Length (bp)	Sequence Length Screened (bp; Consensus Sequences)	Number of Unique Readable Sequences Passed the Screening	Number of Unique Plant Species	Sequence Quality Score (Mean ± SE) *
	Forward	Reverse					
1n1a-ref	347,901	347,901	35–250	>150	65	11	34.37 ± 0.11 ^ac^
1n1b	223,629	223,629	35–250	>100	3	3	34.11 ± 0.12 ^ac^
1n2	117,181	117,181	35–250	>100	1	1	33.53 ± 0.12 ^bc^
1n3	176,223	176,223	35–250	>100	9	3	33.59 ± 0.11 ^c^
1n4	157,468	157,468	35–250	>100	19	12	33.95 ± 0.10 ^c^
1n5	169,266	169,266	35–250	>100	7	3	33.67 ± 0.09 ^bc^
1n6	138,802	138,802	35–250	>100	5	3	33.84 ± 0.10 ^bc^
1n7	163,404	163,404	35–250	>100	11	6	34.11 ± 0.08 ^ac^
1n8	160,706	160,706	35–250	>100	3	1	34.53 ± 0.11 ^a^
1n9	195,474	195,474	35–250	>100	7	5	34.46 ± 0.14 ^a^
1n10	198,622	198,622	35–250	>100	4	3	34.43 ± 0.16 ^ac^

* The mean sequence quality scores that do not share a letter are significantly different at α = 0.05.

**Table 4:** The species identity, origin, and life form of all the ingested plants obtained from the gut contents of the first nymphal instars of *Lycorma delicatula*. Both reference sample and bulk samples are included.

	**Plant Family**	**Plant Species**	**Common** **Name**	**Seq. Length (bp)**	**Highest Match, %**	**Plant Origin**	**Plant Life Form**	**Presence at the Site**	Reported As a Host *
1	Asteraceae	*Ageratina altissima*	White snakeroot	285	99.65	Native	Herbaceous Perennial	−	−
2	Asteraceae	*Mikania scandens*	Climbing hempvine	225	99.55	Native	Woody Vine	−	−
3	Betulaceae	*Betula* sp.	Birch	406	100	Both	Tree	−	+
4	Brassicaceae	*Cardamine impatiens*	Narrowleaf bittercress	258	100	Introduced	Herbaceous Biennial	−	−
5	Caprifoliaceae	*Lonicera maackii*	Amur honeysuckle	247	100	Introduced	Shrub	−	+
6	Celastraceae	*Celastrus orbiculatus*	Oriental bittersweet	404	100	Introduced	Woody Vine	+	+
7	Cornaceae	*Nyssa sylvatica*	Blackgum	362	100	Native	Tree	−	+
8	Fabaceae	*Gleditsia triacanthos*	Honeylocust	260	99.2	Native	Shrub	−	−
9	Fabaceae	*Medicago sativa*	Alfalfa	184	100	Introduced	Herbaceous Perennial	−	+
10	Fagaceae	*Quercus coccinea*	Scarlet oak	336	99.11	Native	Tree	−	+
11	Hydrangeaceae	*Hydrangea* sp.	Hydrangea	115	100	Both	Shrub	−	−
12	Juglandaceae	*Carya illinoinensis*	Pecan	367	100	Native	Tree	−	+
13	Lauraceae	*Litsea* sp.	Litsea	260	99.6	Native	Shrub	−	−
14	Magnoliaceae	*Liriodendron tulipifera*	Tuliptree	292	99	Native	Tree	+	+
15	Moraceae	*Maclura pomifera*	Osage-orange	190	100	Native	Tree	−	−
16	Moraceae	*Morus alba*	White mulberry	357	99.72	Introduced	Tree	−	+
17	Pinaceae	*Pinus strobus*	Eastern white pine	239	100	Native	Tree	−	+
18	Poaceae	*Agrostis stolonifera*	Creeping bentgrass	158	100	Introduced	Herbaceous perennial	−	−
19	Poaceae	*Anthoxanthum odoratum*	Sweet vernalgrass	150	100	Introduced	Herbaceous Perennial	−	−
20	Poaceae	*Festuca* sp.	Fescue	206	100	Introduced	Herbaceous Perennial	+	+
21	Poaceae	*Hierochloe odorata*	Sweetgrass	218	100	Native	Herbaceous Perennial	−	−
22	Poaceae	*Lolium arundinaceum*	Tall fescue	346	99.7	Introduced	Herbaceous Annual	−	−
23	Poaceae	*Milium effusum*	American milletgrass	110	100	Native	Herbaceous Perennial	−	−
24	Poaceae	*Polypogon monspeliensis*	Annual rabbitsfoot grass	127	99.21	Introduced	Herbaceous Annual	−	−
25	Rosaceae	*Prunus* sp.	Plum	200	100	Native	Tree/Shrub	+	+
26	Rosaceae	*Rosa* sp.	Rose	259	100	Introduced	Woody Vine	+	+
27	Simaroubaceae	*Ailanthus altissima*	Tree of heaven	300	99.67	Introduced	Tree	+	+

* Reported in the latest published host plant list for *L. delicatula* [9] and in the recent findings on ingested plants from *L. delicatula* gut contents [14]; "+": reported, "−": not reported.

## Data Availability

All the data obtained and used during this study are deposited to https://github.com/alina42/SLF-1st-instar (accessed on 25 April 2022). This project is licensed under the terms of the MIT license.

## Supplementary Materials

Raw sequences and sequence quality score data are deposited to https://github.com/alina42/SLF-1st-instar (accessed on 25 April 2022). This project is licensed under the terms of the MIT license.
